# RND1 regulates migration of human glioblastoma stem-like cells according to their anatomical localization and defines a prognostic signature in glioblastoma

**DOI:** 10.18632/oncotarget.26082

**Published:** 2018-09-18

**Authors:** Sabrina Boyrie, Caroline Delmas, Anthony Lemarié, Vincent Lubrano, Perrine Dahan, Laure Malric, José Luis, Julia Gilhodes, Marie Tosolini, Laetitia Mouly, Maxime Lehmann, Christine Toulas, Elizabeth Cohen-Jonathan Moyal, Sylvie Monferran

**Affiliations:** ^1^ INSERM UMR1037, Cancer Research Center of Toulouse, Oncopole, Toulouse, France; ^2^ Institut Claudius Regaud, IUCT-O, Toulouse, France; ^3^ Université Toulouse III, Toulouse, France; ^4^ INSERM UMR825, Université Toulouse III, Toulouse, France; ^5^ Service de Neurochirurgie, Centre Hospitalier de Purpan, Université Toulouse III, Toulouse, France; ^6^ Centre de Recherche en Oncologie biologique et Oncopharmacologie (CRO2), INSERM UMR 911, Aix-Marseille Université, Marseille, France; ^7^ UMR 7213 CNRS, Laboratoire de Biophotonique et Pharmacologie, Tumoral Signaling and Therapeutic Targets, Université de Strasbourg, Faculté de Pharmacie, 67401 Illkirch, France

**Keywords:** glioblastoma stem-like cells, periventricular zone, migration, prognostic signature, RND1

## Abstract

Despite post-operative radio-chemotherapy, glioblastoma systematically locally recurs. Tumors contacting the periventricular zone (PVZ) show earlier and more distant relapses than tumors not contacting the PVZ. Since glioblastoma stem-like cells (GSCs) have been proposed to play a major role in glioblastoma recurrence, we decided to test whether GSC migration properties could be different according to their anatomical location (PVZ+/PVZ–). For that purpose, we established paired cultures of GSCs from the cortical area (CT) and the PVZ of glioblastoma patient tumors. We demonstrated that PVZ GSCs possess higher migration and invasion capacities than CT GSCs. We highlighted specific transcriptomic profiles in PVZ versus CT populations and identified a down-regulation of the RhoGTPase, *RND1* in PVZ GSCs compared to CT GSCs. Overexpression of RND1, dramatically inhibited PVZ GSC migration and conversely, downregulation of *RND1* increased CT GSC migration. Additionally, transcriptomic analyses also revealed a down-regulation of *RND1* in glioblastoma compared to normal brain. Using the glioblastoma TCGA database, low levels of *RND1* were also shown to correlate with a decreased overall survival of patients. Finally, based on signaling pathways activated in patients with low levels of *RND1*, we identified an *RND1*^*low*^ signature of six genes (MET, LAMC1, ITGA5, COL5A1, COL3A1, COL1A2) that is an independent prognostic factor in glioblastoma. These findings contribute to explain the shorter time to progression of patients with PVZ involvement and, point out genes that establish the *RND1*^*low*^ signature as key targets genes to impede tumor relapse after treatment.

## INTRODUCTION

Despite combined modality treatment, including surgery and radio-chemotherapy, the prognosis of patients with glioblastoma remains extremely poor [1]. Almost all the patients will die of a relapse in radiation fields or away from the radiation fields, in the brain parenchyma [2]. The failure of initial therapies, which is mainly dependent on tumor heterogeneity, is crucial in glioblastoma [3, 4]. In an attempt to classify diffuse gliomas, subgroups have been defined based on gene expression profiles and genetic and epigenetic alterations [5]. Moreover, high-resolution genome-wide studies have revealed that multiple clones harbouring a variety of genetic alterations coexist within the same tumor [6]. Molecular heterogeneity exists even at cellular level between cells that carry similar genetic alterations and is induced by environmental factors [3, 4]. Expanding single glioblastoma cells into clonal populations demonstrated unique properties including proliferation, differentiation, and different sensitivities to chemotherapeutic drugs [3]. Finally, among patients with proneural tumor, an increased tumor heterogeneity was correlated to a decreased survival [4].

A subset of glioma cells called glioma stem-like cells (GSCs) form heterogeneous glial tumors. They are responsible for the development and the maintenance of tumors [7] and have been proposed to be responsible for tumor recurrences. GSCs display higher resistance to conventional radio-chemotherapy treatments than non GSCs [8, 9]. After treating mice with temozolomide, an alkylating drug currently used in standard glioblastoma treatment, GSCs can still form new tumors [10]. GSCs preferentially reside in perivascular niches where they interact and communicate with tumor associated endothelial cells, via their basement membrane [11]. When orthotopically xenografted, GSCs form tumors that recapitulate the phenotype of patient tumors, notably the ability of glioblastoma cells to infiltrate diffusely [12]. Finally, GSCs are more invasive than their differentiated progeny cells [13].

To invade, glioma cells must initiate dynamic changes in the cytoskeleton organization, notably via integrins [14]. Integrins specifically bind to extracellular matrix proteins, connect their cytoplasmic domain to cytoskeleton and signalling proteins. In this way, integrins control RhoGTPases and actin reorganization that leads to cell migration. Among integrins expressed in glioblastoma cell lines, αvβ3 and αvβ5 integrins are involved in glioma invasion and progression to high-grade glioma [15]. The fibronectin receptor, α5β1 integrin, has also been shown as a promising therapeutic target for high-grade glioma [16]. Besides, elevated expression of α6 integrin from samples of glioblastoma patients is also correlated with a poor patient prognosis [17]. Two studies demonstrate that GSCs overexpressed α6β1 and a3β1 integrins in comparison to non-GSCs [17, 18] suggesting a role of these laminin receptors in GSC invasion. A better understanding of GSC migration toward their microenvironment, notably through integrin involvement, may influence the development of more effective therapies for glioblastoma.

In relation with tumor heterogeneity, clinical data, including ours, demonstrates that relapses in glioblastoma after radio-chemotherapy are more aggressive in tumors contacting the PVZ (PVZ+) than in PVZ– tumors. PVZ+ patients progress quicker and have a decreased overall survival compared to those with tumors not contacting the PVZ (PVZ–) [19–21]. Moreover, we have also recently shown that contact of glioblastoma with PVZ was an independent prognosis factor of shorter progressive free survival after re-irradiation [22]. We hypothesized that the high capacity of PVZ+ tumors to diffusely infiltrate could be explained by a highest migration capacity of GSCs of PVZ+ tumor in comparison to GSCs from the cortical zone (CT). To investigate this, we established GSC paired cultures from CT and PVZ tumor part for two patients diagnosed with glioblastoma. We demonstrated that gene expression hallmarks of GSCs are dependent on their anatomical origin and that PVZ GSCs possess higher migration capacities than CT GSCs. Among migration genes differentially expressed between CT and PVZ GSCs, we showed that overexpression of the RhoGTPase, RND1, dramatically inhibited PVZ GSC migration and, downregulation of RND1 increased CT GSC migration. *In silico* analysis showed that low expression of *RND1* constitutes a bad prognosis factor for glioblastoma patients. Finally, based on signaling pathways activated in patients with low levels of *RND1*, we identified six genes that define an RND1^low^ signature that is an independent prognostic factor in glioblastoma.

## RESULTS

### Characterization and molecular heterogeneity of GSCs derived from CT and PVZ

**Figure 1:**
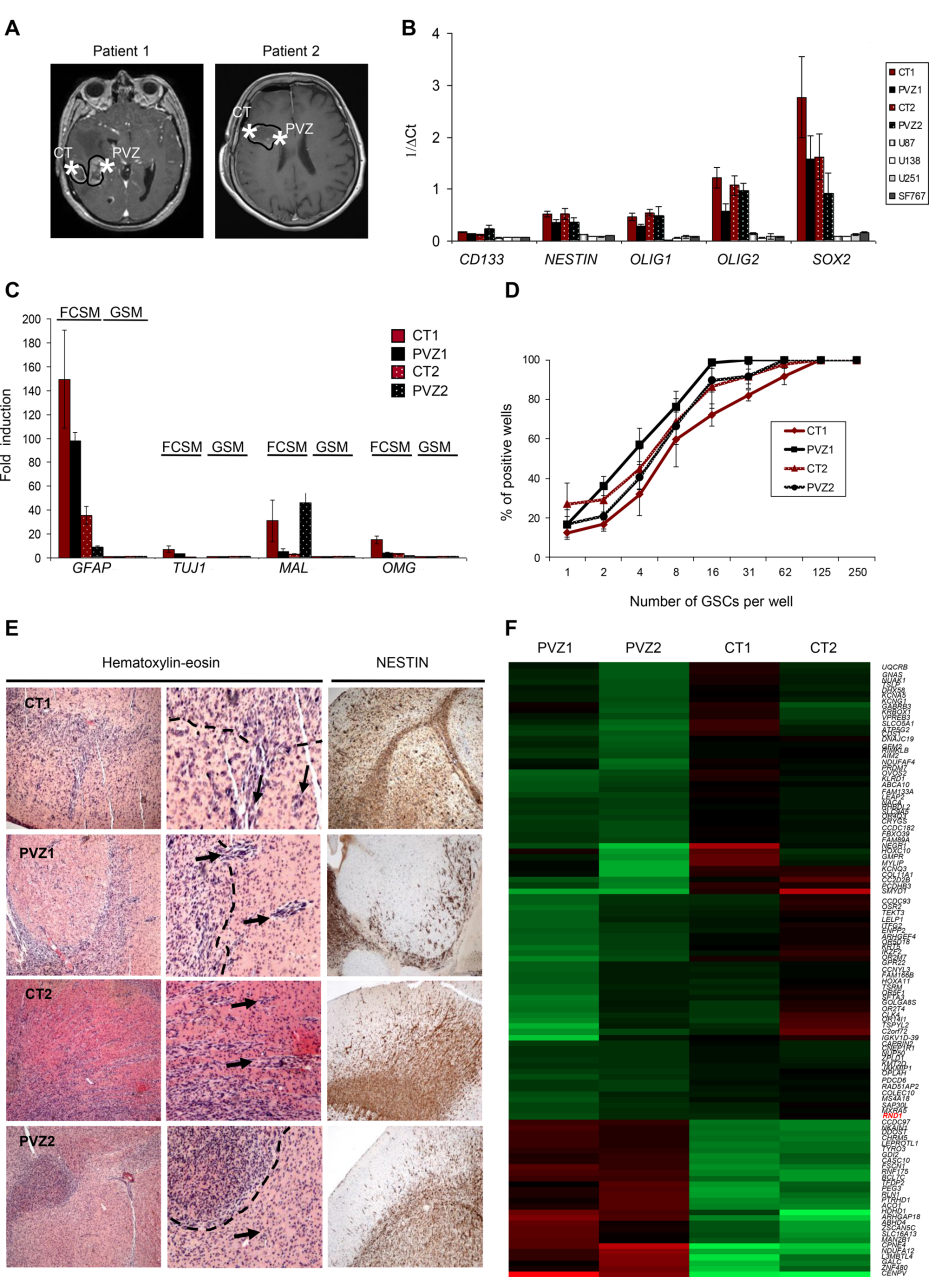
Characterization of GSCs derived from CT and PVZ (**A**) Representative Gadolinium enhanced T1-weighted magnetic resonance imaging showing a right-sided glioblastoma for patient 1 and patient 2. Star symbols mark the location of the extracted tissue sample from the CT or the PVZ. (**B**) The expression of glioblastoma stem cell markers (*CD133*, *NESTIN*, *OLIG1*, *OLIG2* and *SOX2*) was analyzed in GSC neurospheres (CT1, PVZ1, CT2 and PVZ2) or in U87, U138, U251, SF767 cells by RT-qPCR. Data is shown as means of 1/∆Ct (±SEM) from at least 3 experiments. (**C**) The expression of cell differentiation markers (*GFAP*, *TUJ1*, *MAL* and *OMG*) was analyzed by RT-qPCR in GSCs cultured in glioblastoma stem cell medium (GSM) or cultured in medium with 10% of FCS (FCSM). Data is the fold inductions expressed as means of fold induction (±SEM) of at least 3 independent experiments compared with the related control (CT and PVZ cultured in GSM). (**D**) CT1, PVZ1, CT2, PVZ2 GSCs were seeded in 96-well plates at different low cell densities (1 to 250 cells/well) to study their ability to generate secondary neurospheres through limiting dilution assays. The results from 3 experiments are expressed as percentage of positive wells (means ± SEM). (**E**) Hematoxylin and eosin staining (left and center panel, center: enlarged pictures: ×4) and nestin immunostaining (right panel) of mice brains orthotopically xenografted with GSCs. Dotted lines: core tumor. Arrows: invasive extensions as well as disseminated tumor clusters. (**F**) Gene expression analysis of mRNA of GSC neurospheres was performed on an Affymetrix Human Gene 2.0 ST array. Heat map generated from this microarray data showing differentially expressed genes between CT and PVZ GSCs: upregulated (red) or down-regulated (green) genes.

Glioblastoma samples from two patients were removed from enhanced contrast regions on MRI in the CT and in the PVZ (Figure 1A). We established paired cultures of CT and PVZ GSCs (CT1, PVZ1, CT2, and PVZ2) and analyzed their stemness properties. CT and PVZ cells expressed neural tumor stem cell markers *CD133, NESTIN, OLIG1, OLIG2, SOX2* and *A2B5* [23] (Figure 1B, Supplementary Figures 1A and 1B). After culture in DMEM-F12 supplemented with 10% FCS (FCSM), CT and PVZ GSCs were able to differentiate into neuronal-like and astrocytic-like cells (Supplementary Figure 1C) and to express differentiation markers (*GFAP*, *TUJ1*, *MAL* and *OMG*) [23] (Figure 1C). We established with a classic limiting dilution assay that CT and PVZ neurospheres gave rise to secondary neurospheres (Figure 1D and Supplementary Table 1). Both CT and PVZ GSCs had the ability to form diffusely infiltrated tumors when xenografted in nude mice brain (Figure 1E). Altogether, our data shows that CT and PVZ cells derived from our patient samples possess GSC characteristics.

To determine whether PVZ GSCs possess a specific genomic signature, we performed a gene expression microarray. As shown on Figure 1F, different patterns of gene expression have been obtained according to the initial tumor location of the GSCs. We identified 108 genes differentially expressed between CT and PVZ cells (*p* < 0.001), associated with essential biological functions including cell adhesion, apoptosis, transcription and metabolic process. Up-regulated genes in PVZ GSC included RhoGTPase activating protein 18, transcription factor DP-2 and mannosidase alpha whereas down-regulated genes in PVZ cells included collagen type XI-alpha1, RhoGTPase, *RND1* and protocadherin beta3 (Supplementary Table 2). Besides these protein coding genes, CT and PVZ cells differ in the expression of gene expression regulators (antisense RNA, miRNA, long intergenic RNA) and of regulators that guide chemical modifications of others RNAs (small nucleolar RNA…) (Supplementary Table 3). These results demonstrate the molecular heterogeneity of GSCs according to their brain tumor location, notably in the migration processes.

### Invasion ability is increased in PVZ GSCs compared to CT GSCs

**Figure 2:**
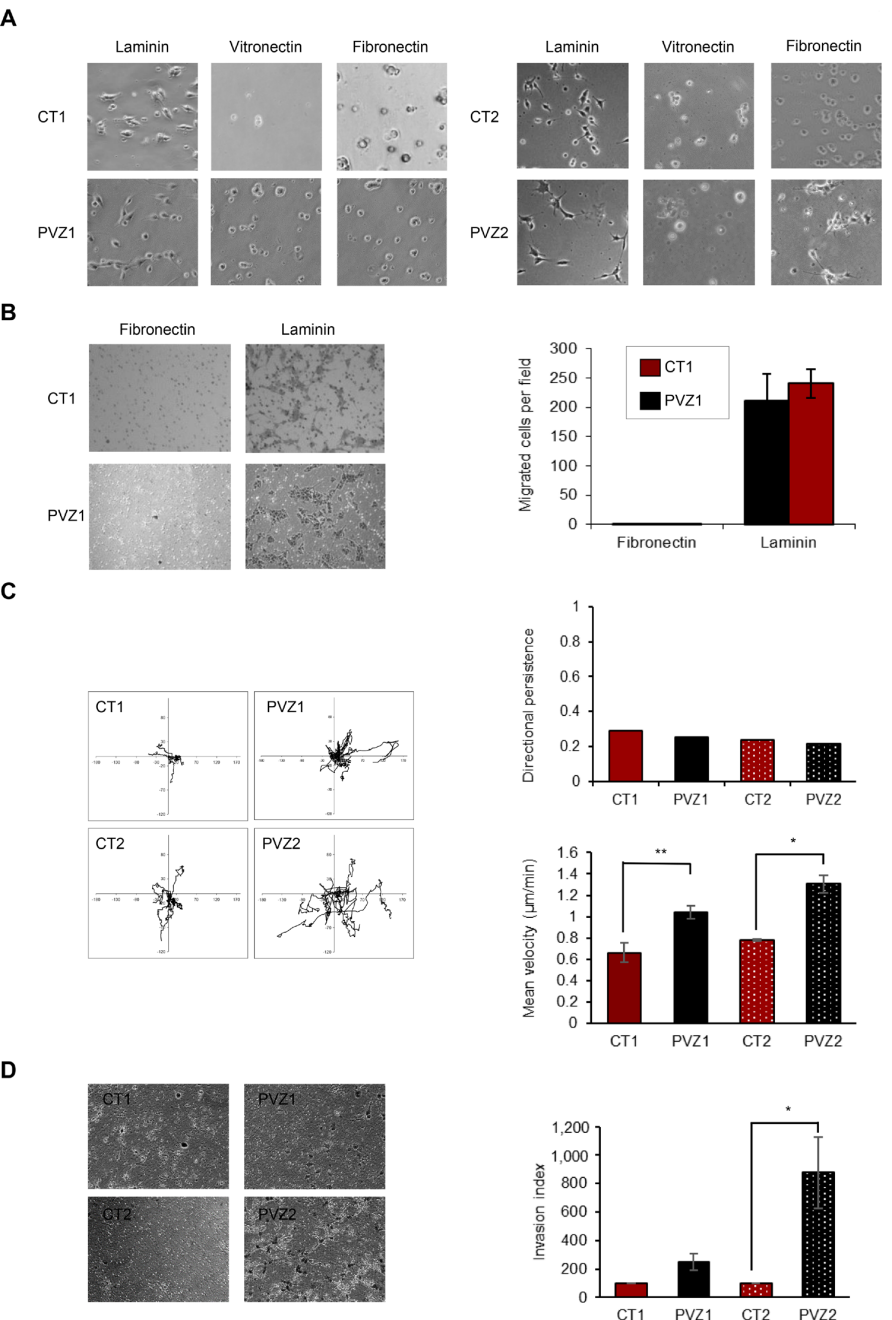
Invasion ability is increased in PVZ GSCs (**A**) CT1, PVZ1, CT2, PVZ2 GSCs were seeded on the indicated extracellular matrix proteins and then allowed to spread for 3 hours. Then, phase–contrast photographs were taken under ×10 magnification. (**B**) CT1 and PVZ1 GSCs were seeded in the upper reservoir of Transwells coated on their undersurface with fibronectin or laminin, and then cells were allowed to migrate into the lower chamber for 24 h. Migrated cells were fixed, stained and counted. Left: representative pictures. Right: Data is shown as means (±SEM) from 3 experiments performed in duplicate. (**C**) Migration of individual CT1, PVZ1, CT2, PVZ2 cells was recorded by time-lapse videomicroscopy over 4 h at 37° C. Left panel: As illustration, migration paths of 8 cells followed during 4 h are represented for each condition. Right panels: Top: One representative experiment of directional persistence. Bottom: Cell velocity was quantified (µm/min) as described in Materials and Methods. Mean cell velocity is expressed as means (±SEM). *n* = 3 (at least 30 individual cells per condition per experiment were analyzed) ^*^p < 0.05; ^**^p < 0.02. (**D**) CT and PVZ cells were seeded in the upper reservoir of Transwell chambers coated with growth factor reduced Matrigel in pure DMEM-F12 medium and allowed to invade for 48 h. Following invasion, non-invading cells were removed from the top chamber and invading cells were fixed, stained and counted. Left Representative photographs of invading cells in a field. Right: Data is shown as means of invasion index (±SEM). *n* = 2 for CT1 and PVZ1 cells; *n* = 4 for CT2 and PVZ2 cells. ^*^p < 0.05.

Cell spreading is the first step of cell invasion. GSCs reside preferentially in perivascular niches and interact with brain blood vessel basement membrane [24]. First, to compare CT and PVZ GSCs migration properties, we performed spreading assays on laminin, fibronectin and vitronectin, three extracellular matrix proteins found in the basement membrane of brain blood vessels and involved in glioma pathogenesis [15–17]. We showed that laminin is a critical extracellular matrix protein for CT and PVZ GSCs and that there was no difference of cell spreading on laminin according to the tumor location (Figure 2A and Supplementary Figure 2A). To further characterize migration properties of these GSCs, we performed directional migration assay in Transwells coated on their undersurface with fibronectin or laminin. None of the GSCs was able to migrate toward fibronectin whereas they all successfully migrated toward laminin (Figure 2B). No significant difference in GSC haptotaxis toward laminin was observed regardless of their initial location in the brain. To sharpen the characterization of GSC migration, we performed time-lapse videomicroscopy of single GSC seeded on laminin. Quantification of single cell migration revealed that PVZ GSCs migrated significantly faster than CT GSCs -as shown by the mean velocity determination-demonstrating differential migration capacities according to the original tumor location (Figure 2C). This result shows that PVZ GSCs have a higher capacity to explore their environment and to scatter into it than CT GSCs. All the GSCs migrated in different directions over the entire surface and no difference in directional persistence was observed according to the tumor location (Figure 2C). Finally, to assess the invasion ability of GSCs, we performed invasion assays in Transwells coating with growth factor reduced Matrigel. Figure 2D shows that PVZ cells invaded more than CT cells. These results showed that laminin is a permissive substrate for CT and PVZ GSC migration and that some GSC invasion properties are dependent on their location in the brain.

### RND1 is down-regulated in PVZ GSCs in comparison to CT GSCs

**Figure 3:**
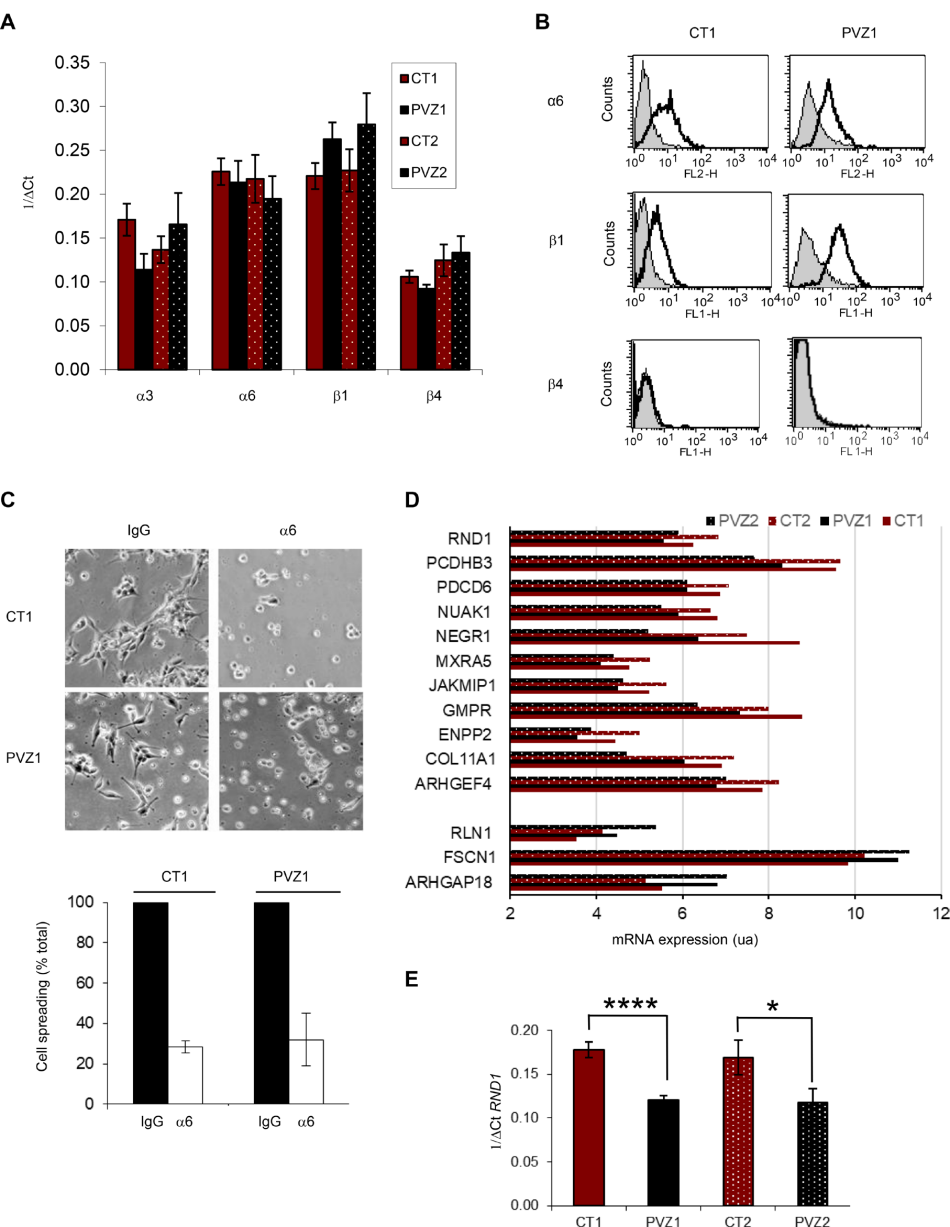
RND1 is down-regulated in PVZ GSCs in comparison to CT GSCs (**A**) Expression of a3, α6, b1 and β4 integrins in GSC neurospheres by RT-qPCR. Data is shown as means (±SEM) from at least 3 experiments. (**B**) Expression of α6, b1 and β4 integrins in CT1 and PVZ1 GSCs by FACS. Grey profiles: isotypic control. Black profiles: specific integrin antibody. (**C**) CT1 or PVZ1 GSCs were pre-incubated with a function-blocking antibody against α6 subunit (α6) or isotypic control (IgG) for 30 minutes at 37° C and then, cells were plated on laminin for 1 hour. Top: representative pictures are shown. Bottom: data is shown as means (±SEM) from at least 3 experiments. (**D**) Differential expression of adhesion/migration genes from GSC neurospheres was performed with an Affymetrix Human Gene 2.0 ST array. Data is shown as means of mRNA expression of indicated gene normalized with the RMA method. (**E**) Expression of *RND1* in GSC neurospheres (CT1, PVZ1, CT2 and PVZ2) was analyzed by RT-qPCR. Data is shown as means (±SEM) from at least 3 experiments. ^*^p < 0.05; ^****^p < 0.001.

To explain why PVZ GSCs migrate significantly faster than CT GSCs on laminin, we first determined the expression of the main receptors for laminin in glioma cells, ie α6β1, α6β4 and a3β1 integrins [18, 25]. By RT-qPCR, we showed that α6 integrin and β1 integrin mRNAs were strongly expressed in GSCs whereas α3 integrin and β4 integrin mRNAs were weakly expressed (Figure 3A). FACS analyses determined that CT and PVZ GSCs expressed α6 integrin and β1 integrin at high levels on their surfaces, and did not express β4 integrin (Figure 3B). Integrin expression may slightly differ between GSC lines, but independently of the initial tumor location of GSCs. In order to determine whether the integrin involved in laminin GSCs spreading could be different between CT and PVZ cells, we performed cell-spreading assays using functional blocking antibodies. As shown in Figure 3C, inhibition of α6 integrin significantly decreased CT1 and PVZ1 GSC spreading in a similar manner. Besides integrins, we highlighted in our gene expression microarray fourteen genes, including RND1, known to be involved in adhesion/migration that were differentially expressed between CT and PVZ cells (Figure 1F). Of the fourteen significantly altered genes, three were up-regulated and eleven were down-regulated in PVZ GSCs (Figure 3D). *RND1* expression is significantly down-regulated in PVZ GSCs in comparison to CT GSCs (pPVZ1/CT1: 0.000019; pPVZ2/CT2: 0.00000497). RND1 is an atypical RhoGTPase that decreases cell adhesion via the inhibition of the formation of actin stress fibers [26]. As RhoGTPases are known regulators of cell migration and have been recently demonstrated as key elements of glioma pathogenesis [27], we focused our study on the role of RND1 in GSC migration. By RT-qPCR, we confirmed the significant down-regulation of *RND1* mRNA in PVZ GSCs in comparison to CT GSCs (Figure 3E). To conclude, PVZ GSCs possess both lower levels of *RND1* and higher migration properties in comparison to CT GSCs.

### RND1 suppresses GSC spreading and migration towards laminin

**Figure 4:**
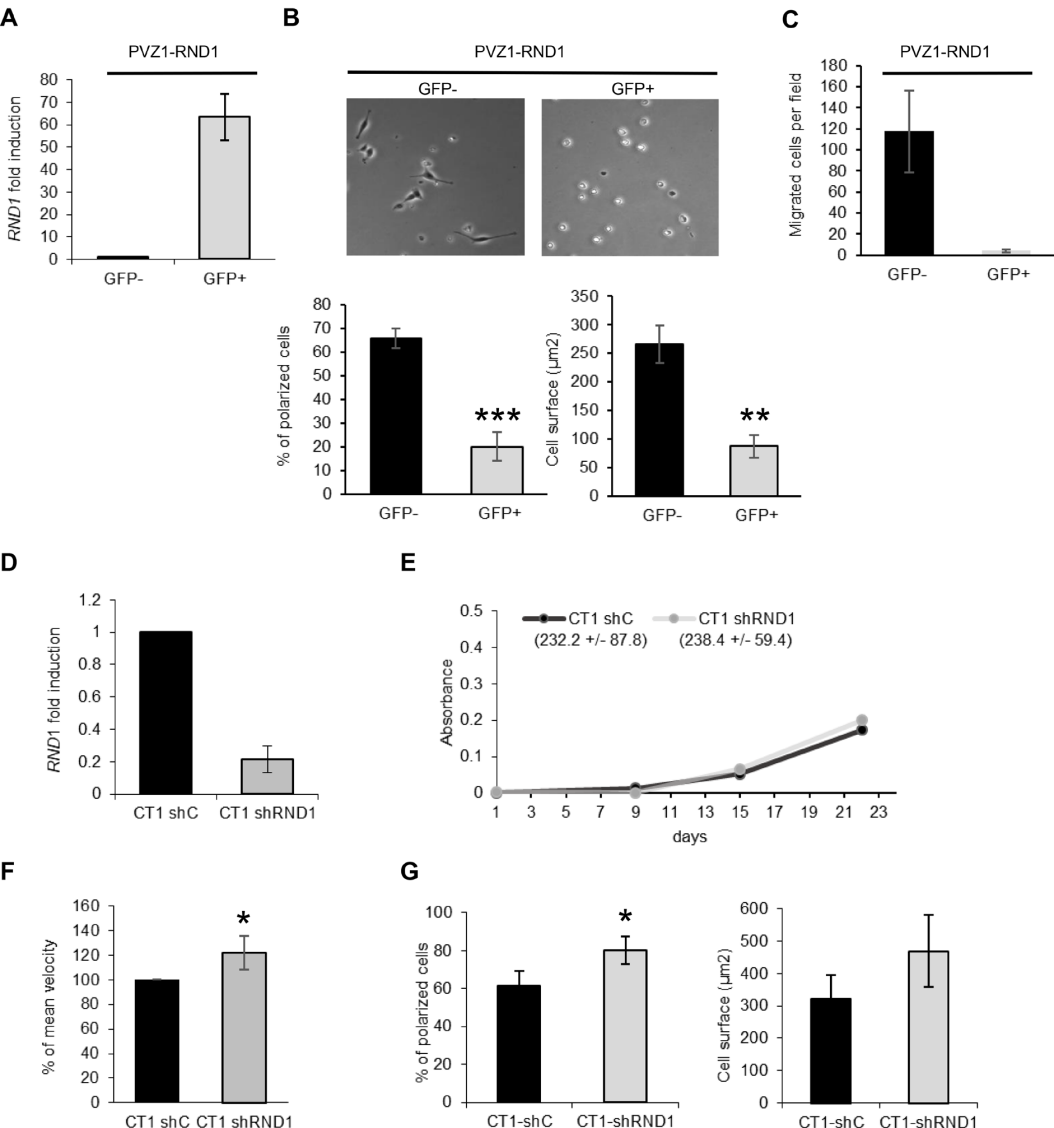
RND1 suppresses spreading and migration abilities of GSCs (**A**) PVZ1 GSCs were transfected with a plasmid encoding a fusion protein of EGFP and RND1 (PVZ1-RND1 cells). After cell sorting, the expression of *RND1* in GFP positive and negative PVZ1-RND1 cells was analyzed by RT-qPCR. (**B**) GFP negative (GFP-) and GFP positive (GFP+) PVZ1-RND1 cells were seeded on laminin, then allowed to spread for 3 h. Top: Phase-contrast photographs were taken under ×10 magnification. Bottom: In each experiment, the cell surface and the percentage of polarized cells were analyzed (at least 30 individual cells per condition per experiment were analyzed). Bars represent means (±SEM) from 3 experiments performed in duplicate; ^**^p < 0.02; ^***^p < 0.01. (**C**) GFP negative (GFP–) or positive (GFP+) PVZ1-RND1 cells were seeded in the upper reservoir of Transwells coated on their undersurface with laminin and then, the cells allowed to migrate into the lower chamber for 24 h at 37° C. Migrated cells were fixed, stained and counted. Data shown as means (±SEM) from 3 experiments performed in duplicate. (**D**) CT1 cells were stably transduced with lentiviral particles expressing a shRNA directed against RND1 (CT1 sh*RND1*) or a control shRNA (CT1 shC). The expression of *RND1* in CT1 shC and CT1 sh*RND1* cells was analyzed by RT-qPCR. Data is shown as fold induction means (±SD) from 6 experiments. (**E**) Viability of CT1 shC and CT1 shRND1 cells was analyzed by a WST-1 assay. One representative experiment is shown. The Figures in the brackets represent the means of proliferation rate (±SEM) from 3 experiments performed in triplicate. (**F**) Migration of individual CT1 shC and CT1 sh*RND1* cells plated on laminin was recorded by time-lapse videomicroscopy over 4 h at 37° C. The mean cell velocity of CT1 sh*RND1* is compared to the mean cell velocity of CT1 shC used as a reference. ^*^p < 0.05. (**G**) CT1 shC and CT1 sh*RND1* cells were seeded on laminin, then allowed to spread for 1 h. In each experiment, the cell surface and the percentage of polarized cells of at least 30 individual cells were analyzed. Bars represent means (±SEM) from 3 experiments performed in duplicate; ^*^p < 0.05.

To test a potential correlation between *RND1* low-level expression and PVZ GSCs high migration, we investigated whether high levels of RND1 protein in PVZ cells could decrease their migration ability. PVZ1 cells were transfected with a plasmid encoding a fusion protein of EGFP and RND1 (PVZ1-RND1) or with a plasmid encoding EGFP (PVZ1-EGFP). GFP positive or GFP negative GSCs were selected by FACS (Supplementary Figure 3A). We confirmed *RND1* overexpression in GFP positive PVZ1-RND1 GSCs by RT-qPCR (Figure 4A, Supplementary Figure 3B), which did not affect the cell viability (Supplementary Figures 3D and 3E). We then showed that, three hours after seeding on laminin, GFP positive PVZ1-RND1 cells remained round and stayed in suspension whereas GFP negative PVZ1-RND1 cells spread on laminin (Figure 4B) like the control cells (Supplementary Figure 3C). Furthermore, overexpression of RND1 in PVZ1 cells dramatically decreased their ability to migrate toward laminin after 24 h (Figure 4C). We next investigated the consequence of RND1 loss on migration in CT1 cells. CT1 cells were transduced with lentiviral particles expressing a shRNA directed *RND1* (CT1 sh*RND1*) or a control shRNA (CT1 shC). *RND1* expression was down regulated in stably transduced CT1 sh*RND1* cells in comparison to CT1 shC (Figure 4D). RND1 loss has no effect on cell viability (Figure 4E) whereas it induces a slight but significant increase in mean velocity (Figure 4F) and in cell spreading (Figure 4G). These results demonstrated that RND1 could suppress spreading and migration abilities of GSCs. Together our data indicates that the differential RND1 expression level between PVZ and CT GSCs may explain, at least in part, their different migration profiles.

### Lower expression of *RND1* is correlated with a poor prognosis in patients with glioblastoma

**Figure 5:**
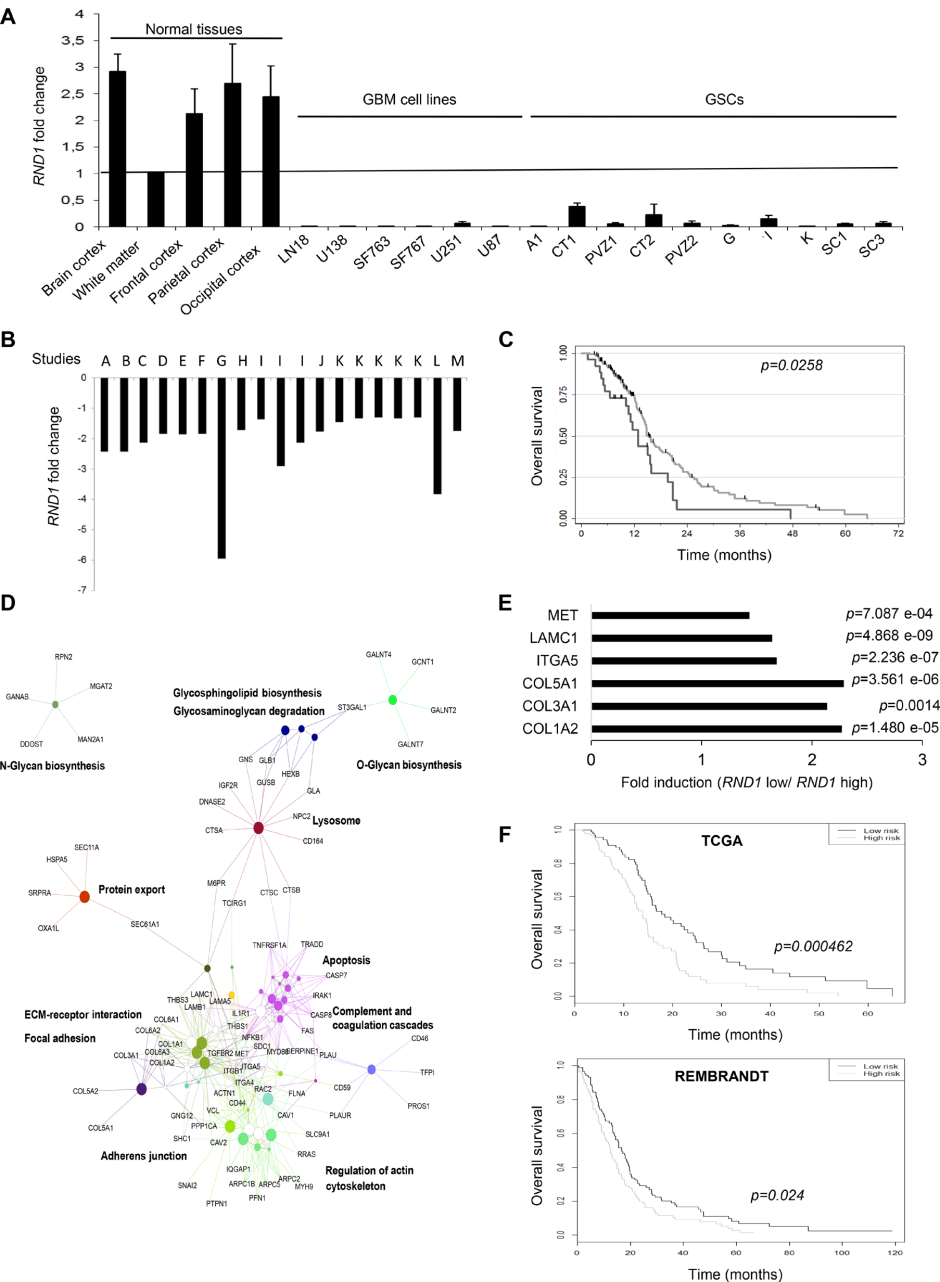
Lower expression of *RND1* is correlated with a worse prognosis in glioblastoma patients and up-regulates the expression of six genes that establish a prognostic signature for glioblastoma (**A**) *RND1* mRNA expression in normal brain tissues, in glioblastoma cell lines (LN18, U138, SF763, SF767, U251, U87) and in GSCs (A1, CT1, PVZ1, CT2, PVZ2, G, I, K, SC1, SC3) was determined by RT-qPCR. *RND1* mRNA levels in the white matter were used as a reference of normal brain expression. Data is shown as fold induction means from at least three experiments. (**B**) *RND1* mRNA expression fold change in glioblastoma samples compared to normal brain tissues from thirteen gene expression datasets, described in Supplementary Table 11 (studies A to M; several datasets are available for studies I and K). (at least *p* < 0.05, described in Supplementary Table 11). (**C**) Kaplan–Meier curves of overall survival for glioblastoma patients with a lower *RND1* expression (black, *n* = 26) or a higher expression (grey, *n* = 158), determined with TCGA database. Log rank *p*-value (down-regulated versus upregulated) = 0.0258. (**D**) Thirteen activating signaling pathways in patients with low *RND1* expression from KEGG analysis. (**E**) The expression of the six genes that constitute the *RND1*^low^ signature determined with TCGA database. Data is shown as fold induction means (mRNA expression means of each gene in patients with low levels of *RND1* relative to patients with high levels of *RND1*). (**F**) Kaplan–Meier curves of overall survival in TCGA cohort (top) or in REMBRANDT cohort (bottom) stratified by the *RND1*^low^ signature high and low risk as detailed in Materials and Methods. TCGA: 92 patients per group. REMBRANDT: 89 patients per group.

As loss of RND1 is involved in GSCs migration from PVZ+ tumors, known to be more aggressive than PVZ– tumors [19–21], we tested whether *RND1* gene expression could be correlated with glioblastoma prognosis. First, we analyzed *RND1* expression by RT-qPCR in normal brain tissues, in several glioblastoma cell lines and in GSCs established in our laboratory. *RND1* expression is significantly down regulated in glioblastoma cells compared to normal tissues (Figure 5A). To pursue our analysis, using gene expression databases in open access, a meta-analysis of *RND1* expression revealed a significant down-regulation of *RND1* in glioblastoma samples versus normal tissues (*p* < 0.05, Figure 5B). Using TCGA database, we next examined whether this down-regulation of *RND1* was related to the prognosis of glioblastoma patients. Patients with a lower expression of *RND1* (*i.e* <=4.8) showed a worse survival than those with a higher expression of *RND1* (HR = 0.59, 95% CI:0.37–0.94, *p* = 0.028) (Figure 5C). Like patients with a higher expression of *RND1*, patients with a lower expression of *RND1* are divided into the different biological clinical parameters (Supplementary Table 4). Interestingly, patients with a lower expression of *RND1* mostly belong to the mesenchymal subtype (Supplementary Table 4). The mesenchymal subtype in glioma is defined by a genomic and transcriptomic profile, notably by higher expression levels of mesenchymal markers such as CHI3L1/YKL40 and MET [28, 29]. To determine whether a lower expression of *RND1* correlates with an overexpression of mesenchymal genes, we performed Spearman’s correlation tests using the TCGA database. Supplementary Figure 4 shows that *RND1* expression is inversely correlated with common mesenchymal genes [30–33] and specific glioma mesenchymal genes [28, 29, 34]. These results clearly demonstrated that a lower expression of *RND1* correlates with a higher expression of mesenchymal genes and, is a factor of poor prognosis for glioblastoma patients.

### *RND*1^low^ signature is an independent prognostic factor in glioblastoma

To determine which signaling pathways controlled by RND1 might be involved in the prognosis of glioblastoma recurrence and thus survival, we performed a functional enrichment of genes. In patients with low expression of *RND1* (*RND1*^low^), we identified thirteen signaling pathways that are activated (Figure 5D and Supplementary Table 5). The most significant being are the “extracellular matrix-receptor interaction” pathway (*p* = 1.32 e-09), the “focal adhesion” pathway (*p* = 1.05 e-07) and the “lysosome” pathway (*p* = 1.72 e-04). This raised the hypothesis that genes from these pathways could be involved in the worse survival prognosis of patients with low *RND1* expression. To assess the relationship with overall survival of *RND1* and genes from “extracellular matrix-receptor interaction” and “focal adhesion” pathways, a penalized cox regression model with lasso selection was used. We identified six prognostic genes -*ITGA5, COL3A1, COL5A1, MET, COL1A2* and, *LAMC1*- (Supplementary Table 6) that we gathered under the name of *RND1*^low^ signature. These genes were all significantly overexpressed in patients with low *RND1* expression compared to patients with high *RND1* expression (Figure 5E). We then calculated the signature risk score for each patient in the TCGA database and, divided them into a high-risk group and a low-risk group by taking the median value of risk score. The median overall survival in the low-risk group is 17.8 months versus 13.8 months for the high-risk group (Figure 5F, top). The *RND1*^low^ signature was significantly associated with overall survival (*p* < 0.001). To validate our prognostic signature, the training model was applied to glioblastoma patients from REMBRANDT database. Consistent with TCGA results, our *RND1*^low^ signature predicts survival of glioblastoma patients (Figure 5F, bottom). Using TCGA, a multivariate cox regression analysis with clinical parameters was carried out to test the strength of *RND1*^low^ signature in its ability to predict survival. This analysis showed that the *RND1*^low^ signature remains a strong prognostic factor, independently of clinical parameters (Table 1, *p* = 0.0042). To conclude, we identified an *RND1*^low^ signature that is an independent prognostic factor in glioblastoma.

**Table 1:** Multivariate Cox regression analysis for RND1low signature and other prognostic markers

Factor	HR	*p* value	IC 95%
Risk score high vs Low	2.22	**0.0042**	[1.29–3.83]
Karnosky ≥70	0.87	0.6643	[0.46–1.63]
Tumor resection vs others	1.85	**0.0401**	[1.03–3.32]
Age ≥60	1.16	0.5459	[0.71–1.91]
Mesenchymal vs Classical	1.49	0.2175	[0.79–2.83]
Neural vs Classical	1.38	0.4012	[0.65–2.90]
Proneural vs Classical	1.58	0.3007	[0.67–3.73]
Non G-CIMP vs G-CIMP	1.20	0.7152	[0.44–3.26]
MGMT methylated vs non methylated	0.50	**0.0049**	[0.31–0.81]

## DISCUSSION

The aim of this work was to establish whether GSC migration heterogeneity exists according to the initial location of these cells within the tumor (PVZ+ or PVZ–). By using an original model of GSCs isolated from CT and PVZ, we demonstrated that PVZ GSCs migrated faster and invaded more than CT GSCs and, that their migration may be controlled by RND1. Moreover, we demonstrated that low-expression of *RND1* in glioblastoma patient samples was correlated with a worse prognosis for patients. Finally, we identified an *RND1*^low^ signature that predicts outcome for glioblastoma patients.

A key finding of our pilot study is that the migration/invasion properties of GSCs depend on their anatomical location. This work will be the foundation for further studies, with a larger number of patients, but shows the increased migration ability of PVZ GSCs compared to CT-derived cells. The increased invasion ability of PVZ GSCs could explain the worse clinical outcome of PVZ+ patients. Only a few studies have previously described different GSC migration abilities according to their location. In fact, a GSC line derived from the PVZ, injected into a mouse brain was shown to invade the corpus callosum and the contralateral hemisphere whereas a GSC line derived from CT was not able to invade these sites [35]. More recently, it has been shown that the GSCs from peritumoral parenchyma are much more invasive than the GSCs from the tumor mass [36]. As CT and PVZ samples come from the same patient, our study illustrates the importance of the intratumoral heterogeneity on tumor behavior. In this study, we investigated and identified genes that may distinguish CT and PVZ GSCs. These genes are involved in cell migration, metabolism, transcription, translation, intracellular traffic, regulation of apoptosis and cell survival (Supplementary Table 2). A recent study analyzed proteins that are significantly altered in PVZ+/PVZ– tissues of glioblastoma patients [37]. In accordance to our results, they found that pathways involved in metabolism (notably in oxidative phosphorylation), extracellular matrix receptor interaction and, migration are affected in PVZ+ glioblastoma. All these functions are hallmarks of cancer and open new lines of research to explain the higher resistance to treatment and the poor overall survival of patients with a glioblastoma contacting the PVZ.

Besides this, we demonstrated in our GSC model that the higher migration ability of PVZ GSCs is potentially associated to lower levels of *RND1*. Only four recent studies explored the role of RND1 in cell migration. Consistent with our present data, inactivation of RND1 induces the invasion of immortalized breast cells in 3D matrigel and, overexpression of RND1 diminishes lung colonization in mice xenografted with breast cancer cells [38]. On the contrary, overexpression of RND1 in esophageal carcinoma cells promotes their migration [39]. This discrepancy concerning the role of RND1 in invasion is correlated with the difference of *RND1* misregulation in these cancers. In fact, *RND1* expression is down-regulated in glioblastoma patients and in the most aggressive subtypes of breast cancers [38] but it is up-regulated in esophageal squamous cell carcinoma [39]. Overexpression of RND1 suppresses focal adhesion sites [26] whose formation and turnover are crucial for cell migration [40]. It is known that there is a bi-phasic migration response to cell adhesion since both too weak and too strong adhesion can reduce cell migration [41]. Our functional enrichment of genes in patient tumors revealed that low expression of *RND1* in glioblastoma induces an overexpression of focal adhesion proteins like extracellular matrix proteins (*COL1A1* and *LAMB1*); integrins (*ITGA5* and *ITGB1*); actin-binding proteins (*FLNA*; *ACTN1*) and vinculin (Supplementary Table 5). Moreover, it was previously shown that overexpression of RND1 in fibroblasts decreases the expression of vinculin [26]. We could hypothesize that misregulation of *RND1* expression in glioblastoma cells leads to an optimal formation and turnover of focal adhesion sites and therefore, increased migration.

Recurrence of glioblastoma is caused by the combination of local invasion and therapy resistance. Using a data-driven approach, it has been recently demonstrated that the expression of members of RhoGTPases family is a key marker of glioma progression [27]. In fact, the overexpression of RND3, another member of RND subfamily, enhances the invasion of glioblastoma and is correlated with a poor prognosis. For its parts, in response to a protein complex containing pleiotrophin, secreted by neural precursor from the PVZ, RhoA signaling is activated in glioma cells and induces the migration of glioma cells to the PVZ [42]. In this study, we showed that low levels of *RND1*, involved in GSC migration, are also related to a decreased overall survival in patients. Moreover, we found that in tumors, a lower expression of *RND1* correlates with an overexpression of mesenchymal genes and that tumors with a lower expression of *RND1* mostly belong to the mesenchymal subtype. The mesenchymal subtype is associated with a poorer survival than other groups of glioblastoma patients [34, 43]. Interestingly, in breast, depletion of RND1 in immortalized mammary epithelial cells promotes epithelial to mesenchymal transition associated notably with disruption of adherens junctions, downregulation of E-cadherin and up-regulation of fibronectin [38]. Altogether, these results suggest that loss of *RND1* in glioblastoma tumors could drive a mesenchymal subtype and hence, could decrease the overall survival in patients.

Based on functional enrichment of genes in glioblastoma patients, we identified six genes whose expression is inversely correlated to *RND1* and that predict the survival of glioblastoma patients. The *RND1*^low^ signature gathered three qualities: it was discovered from a homogeneous population of glioblastoma patients treated with standard radio-chemotherapy; it involves a short list of genes; and it remains a prognostic factor by itself, independently of clinical parameters. Moreover, the predictive power of the *RND1*^low^ signature remains significant for both the training (TCGA) and validation sets (REMBRANDT). Thanks to these qualities, the *RND1*^low^ signature could be useful in clinical practice to predict the survival of glioblastoma patients. The *RND1*^low^ signature could also lead to clinical application to improve glioblastoma treatment through the targeting of genes involved in this signature. Indeed, *ITGA5* and *MET* were found to be key contributors to the *RND1*^low^ signature with their high BSS (Supplementary Table 6). Integrin α5b1 has recently been described as a fine regulator of glioblastoma cell migration [44]. MET and its ligand HGF create an autocrine signaling loop that promotes GSC invasion [45]. In consequence, targeting *ITGA5* or *MET* genes could inhibit the invasive capacity of glioblastoma cells induced by low *RND1* expression and especially the one of PVZ+ cells. In conclusion, our study contributes to explain the shorter time to progression of patients with PVZ involvement and highlights RND1 as a gene involved in glioblastoma heterogeneity. Our study suggests that genes establishing the *RND1*^low^ signature could be interesting targets for optimizing glioblastoma treatment.

## MATERIALS AND METHODS

### Extracellular matrix proteins, antibodies and primers

Extracellular matrix proteins, antibodies and primers are respectively depicted in Supplementary Tables 7, 8 and 9. As previously described [38], commercial antibodies did not show sufficient affinity to allow the detection of endogenous RND1.

### Tumor samples

Before any therapy, glioblastoma samples were obtained after informed consent from patients admitted to the neurosurgery department at Toulouse University Hospital. Tumors were histologically diagnosed as glioblastoma according to WHO criteria. For patients 1 and 2, two tumor samples were removed from the cortical area (CT1, CT2) and from the periventricular zone (PVZ1 and PVZ2) by utilizing stereotactic image-guided sampling. These patients had a large tumor that was in contact with both CT and PVZ. After mechanical dissociation of tumor tissues, cells were seeded at 37° C in a humid atmosphere of 5% CO_2_ in glioblastoma stem cell medium (GSM) composed of DMEM-F12 (Lonza) supplemented with B27 and N2 additives (Invitrogen), EGF (20 ng/mL) and basic FGF (20 ng/mL) (Peprotech). When neurospheres were formed, they were isolated, dissociated with trypsin and cultured as previously described [23]. The percentage of GSCs into the neurosphere was evaluated by flow cytometry [23] (Supplementary Table 10). For other patients (A1, G, I, K, SC1, SC3), only one tumor sample was removed from different brain zones.

### Cell culture and limiting dilution assays

Limiting dilution assays were performed on GSCs [23]. For cell differentiation, GSCs were grown in DMEM-F12 supplemented with 10% FCS (FCSM) for two weeks [23].

Human LN18, U87, U138, U251, SF763 and SF767 glioblastoma cells were maintained in DMEM (Lonza) supplemented with 10% FCS.

### Cell transfection, cell sorting and cell transduction

Five hundred thousand PVZ1 cells per well were seeded in 6-well plates and then transfected using Fugene HD (Promega) with three µg of p-EGFP-*RND1* (Addgene) or of p-EGFP (Clontech). A second transfection was realized seven days after the first transfection to improve gene expression. One week after the second transfection, GFP-positive or GFP-negative GSCs were sorted by FACS. Sorted GSCs were immediately seeded on culture plates to study their ability to migrate or pelleted to quantify RND1 mRNA expression levels.

Twenty five hundred thousand CT1 cells were transduced with lentiviral particles (MOI of 10:1) containing the pLK0.1-neo-CMVtGFP-shRNA plasmid with a sequence directed against *RND1* mRNA or a control sequence (Sigma-Aldrich). Five days after transduction, transduced cells were selected with G418.

To establish PVZ1-RND1 cell lines, 25,000 PVZ1 cells were transduced with lentiviral particles (MOI of 10:1) containing the pLX317-puromycin-RND1 (which contains the cDNA of *RND1*; Sigma-Aldrich) or a control sequence (tGFP; Sigma-Aldrich). Five days after transduction, cells were selected with puromycin.

### RT–qPCR and differential expression analysis

RNA from normal human cortex and white matter were obtained from Biochain, Origene, Clontech, and Agilent. Total RNA extraction, RT-qPCR protocol and ΔCt analysis were previously described [23]. Beta2 microglobulin or actin was used as endogenous control in the ΔCt analysis. Results of RT-qPCR are expressed either in 1/∆Ct values -used to express the levels of mRNA without comparison to a standard- or fold induction -used when we compared the levels of mRNA under a given condition to a standard.

After RT on GSCs RNA (at least three samples per cell line) with biotinylated desoxyribonucleotides, cDNA were hybridized on an Affymetrix Human Gene 2.0 ST array. Then, the DNA complexes were revealed by fluorescent streptavidin. Images were analyzed by Command Console and normalized with RMA method (data were normalized per cell line). Two lists of differentially expressed genes (CT1 *vs* PVZ1 and CT2 *vs* PVZ2) were established using a criteria based on adjusted *p*-value cut off of 0.001 and log2 fold change >0.65 or <-0.65. In the heatmap, we only showed the differentially expressed genes that were common to both lists and had the same directional change.

### Orthotopic xenograft generation and immunohistochemistry

In accordance with ARRIVE guidelines, the French Institution animal ethics committee approval was obtained for the protocols used on animals. Orthotopic human glioblastoma xenografts were established in 4-6 weeks-old female nude mice (Janvier) with 2.5 × 10^5^ cells as previously described [23]. Each GSC line was xenografted at least in three mice. Mice were sacrificed at the appearance of neurological signs. Immunohistochemistry analysis was performed on the excised brains on paraffin-embedded sections (5 μm) [23].

### Immunofluorescence

Immunofluorescence was performed as previously described [46]. For nestin and sox2 stainings, isolated GSCs were seeded on laminin-coated Labtek slides for 24 hours. For β3-tubulin and GFAP stainings, GSC neurospheres were seeded on laminin-coated Labtek slides and were grown in FCSM for five days.

### Cell spreading assays

Fifteen thousand GSCs were seeded on pre-coated wells with extracellular matrix proteins at 1.5 µg/cm^2^ and incubated at 37° C. When mentioned, GSCs were pre-incubated or not with 20 µg/ml of function-blocking antibodies for 30 min at 37° C. Three random fields per well from duplicate wells were pictured under a 10× objective. Cells were manually delineated. Cell surface (A), perimeter (P) and circularity (C = 4π(A/P^2^)) of at least 30 cells per experiment were calculated using the NIS-Elements Advanced Research 3.0 software (Nikon). Cells were classified into two groups: rounded cells and polarized cells as previously described [47].

### WST-1 cell viability assays

CT1 and PVZ1 cells were seeded in triplicate into 96-well microplates at a density of 3,000 cells per well and allowed to form neurospheres. At indicated time, the WST-1 reagent (Roche Diagnostics) was applied for 1 h at 37° C. The formazan dye was quantified at 450 nm using a plate reader (FLUOstar Optima, BMG Labtech). The proliferation rate (PR) was calculated in the exponential phase of cells growth during seven 7 consecutive days: PR = (Absorbance_Day+8_/Absorbance_Day+1_) × 100.

### Directional migration assays

Directional migration assays were performed as previously described [46] except for the following: twenty thousand GSCs per well were seeded in Transwells (24 wells, BD Biosciences) pre-coated on their undersurface with 1.5 µg/cm^2^ of fibronectin or laminin and incubated for 24 h at 37° C.

### Non-directional migration assays

GSCs (0.75 × 10^4^ cells/cm^2^) were seeded on laminin-coated wells (2 duplicate wells per condition) and were allowed to migrate for 4 h at 37° C, 5% CO_2_. Using an inverted Nikon microscope at 10× magnification, two fields per well were imaged and followed at 3 min intervals with a Coolsnap HQ camera (Photometrics, Tucson, AZ). Manual tracking of the nucleus was performed to follow individual cell migration (at least 30 individual cells per condition per experiment) using NIS-Elements AR 3.0 software as previously described [48]. Directional persistence and mean cell velocity were calculated from time-lapse movies as previously described [48].

### Cellular invasion

GSCs were trypsinized, washed with pure DMEM-F12 medium and seeded (100,000 cells/insert) in pure DMEM-F12 on growth factor reduced Matrigel Transwell chambers (8 μm, Corning). The lower wells contain complete GSM. Cells were allowed to migrate for 48 h, non invading cells were removed from upper wells using a cotton swab and invading cells adherent to the bottom of the insert were fixed with 100% methanol and stained with amido black. Invading cells were enumerated by counting the number of cells in 3 distinct fields for each insert under a 10× objective using an optical microscope. Assays were performed in duplicate. The invasion index was calculated with the following formula: (the mean number of invading cells per field for PVZ/ the mean number of invading cells per field for CT) × 100.

### Flow cytometry

Flow cytometry was performed as previously described [49]. To specifically determine integrin expression in GSCs, the gated strategy was based on the previously described protocol [23].

### *RND1* gene expression meta-analysis

For the meta-analysis of *RND1* gene expression, we selected the thirteen gene expression datasets (depicted in Supplementary Table 11) comparing glioblastoma samples to normal tissues available on the NextBio research browser (https://www.nextbio.com/) [50].

### Survival analysis and functional enrichment

For survival analysis, using the glioblastoma database of TCGA (https://genome-cancer.ucsc.edu/), we focused on patients treated with standard radio-chemotherapy for primary glioblastoma, excluding patients with prior glioma history (*n* = 184 patients). Minimum *p*-value approach was used to dichotomize *RND1* expression (if *RND1*>4.8). Overall survival rates were estimated using Kaplan-Meier method and univariate analyses were performed using logrank test. Two-sided *p*-values of less than 0.05 were considered statistically significant.

Using TCGA, two thousand genes that are the most differentially expressed in patients with low and high *RND1* expression were identified by Student’s *t*-test on all available TCGA patients. The *p*-values were adjusted using the Benjamini-Hochberg procedure for multiple testing [51]. These genes (*p*-value cut off of 0.01 and log2 fold change>1.1 or <0.909) were then analyzed for functional enrichment using the Cytoscape (version 3.4.0) plugin ClueGO (version 2.2.5) [52] compared to the KEGG term. A lasso penalized cox regression was used to identify correlations between overall survival and *RND1*, genes from (KEGG_ECM_RECEPTOR) and (KEGG_FOCAL_ADHESION) pathways [53]. A 10-fold cross validation was realized to select the best penalty parameter lambda. Using a resampling approach, bootstrap selection stability (BSS) was computed for each parameter. From genes selected by the lasso procedure, a risk score prediction was created. It is based on the linear predictor given by the model. This score was then dichotomized by taking the median value of the risk score (threshold = 2.28). Thus, two groups were established (poor versus good prognostic) and corresponded to the signature for this dataset. The high-risk group of the *RND1*^low^ signature corresponds to a group with a value superior to the median value of the risk score and, the low-risk group corresponds to a group with a value inferior to the median value of the risk score. To validate our signature, the training model obtained from TCGA was then applied on glioblastoma patients (*n* = 178) from REMBRANDT database (http://www.betastasis.com/glioma/rembrandt/) and a new risk score was obtained. The risk score was dichotomized like mentioned above, by taking the median value of the new risk score (threshold = 3.15).

### Statistical analysis

To compare the average from different experiments, Student’s test was used. Differences were considered statistically significant at *p* < 0.05; risk of 5%.

To examine whether an underexpression of *RND1* correlates with an overexpression of mesenchymal genes, Spearman’s correlation tests were performed on patients treated with standard radio-chemotherapy for primary glioblastoma, excluding patients with prior glioma history.

## SUPPLEMENTARY MATERIALS FIGURES AND TABLES





## Abbreviations

FCSM: medium supplemented with 10% of FCS
GSCs: glioblastoma stem-like cells
GSM: glioblastoma stem cell medium
CT: cortical area
PVZ: periventricular zone
PVZ+: tumors contacting the PVZ
PVZ–: tumors not in contact with the PVZ
