# Supplementary material for: RND1 regulates migration of human glioblastoma stem-like cells according to their anatomical localization and defines a prognostic signature in glioblastoma
Source: Oncotarget. 2018 Sep 18;9(73):33788–803. doi: 10.18632/oncotarget.26082 (PMC6173464; doi:10.18632/oncotarget.26082)
Supplement: Supplementary file 1 [file oncotarget-09-33788-s001.pdf]

RND1 regulates migration of human glioblastoma stem-like cells according to their anatomical localization and defines a prognostic signature in glioblastoma

SUPPLEMENTARY MATERIALS

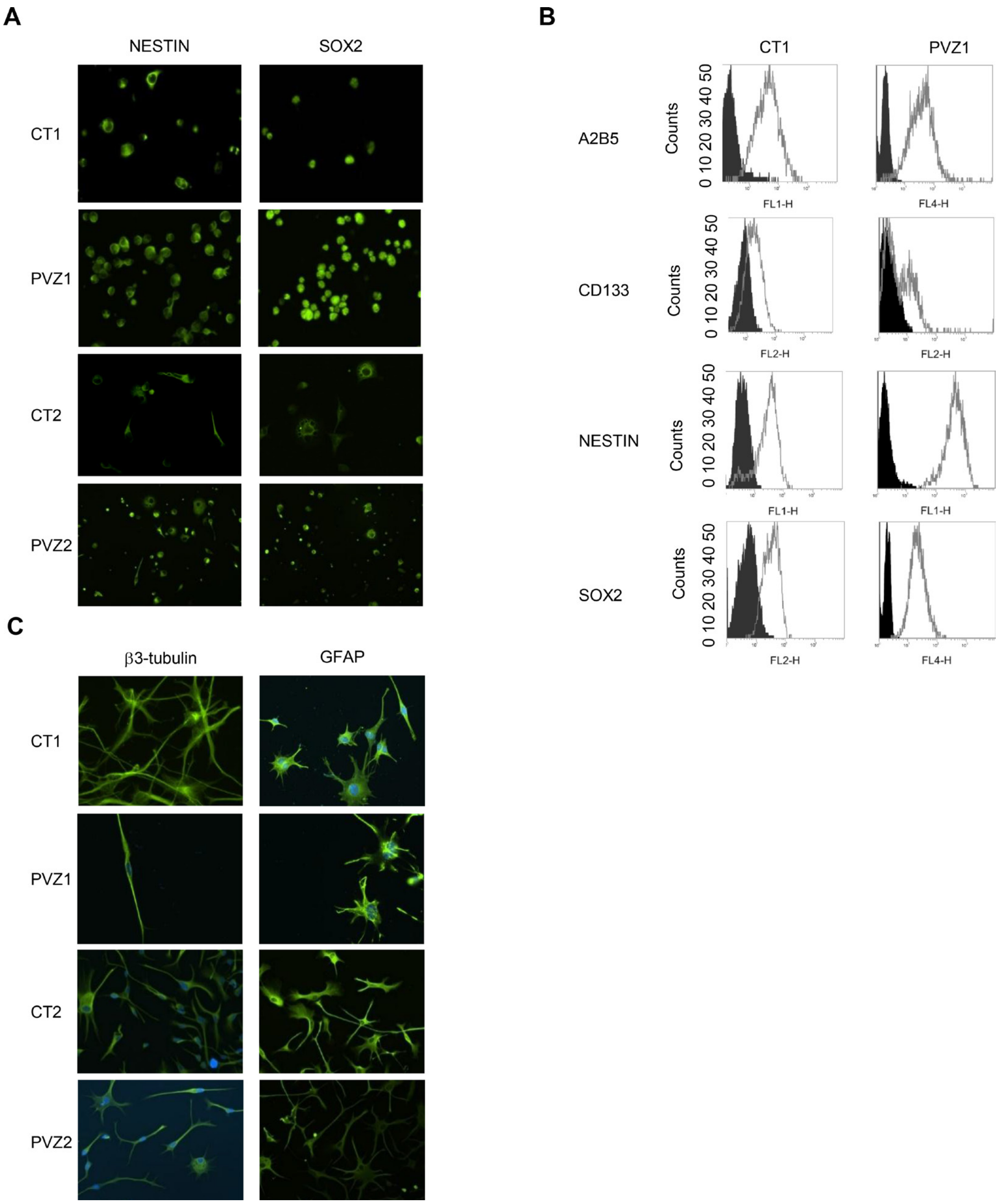

Supplementary Figure 1: Characterization of GSCs. (**A**) Representative images of GSCs seeded on laminin, stained for neural stem cell markers NESTIN and SOX2 (20×). (**B**) Representative FACS analysis of neural stem cell markers in GSCs. Black profiles: isotypic controls. Grey profiles: specific neural stem cell markers antibodies. (**C**) Representative images of GSCs cultured in FCSM for one week which become positive for β3-tubulin, a neuronal marker, and GFAP, an astrocyte marker (20×).

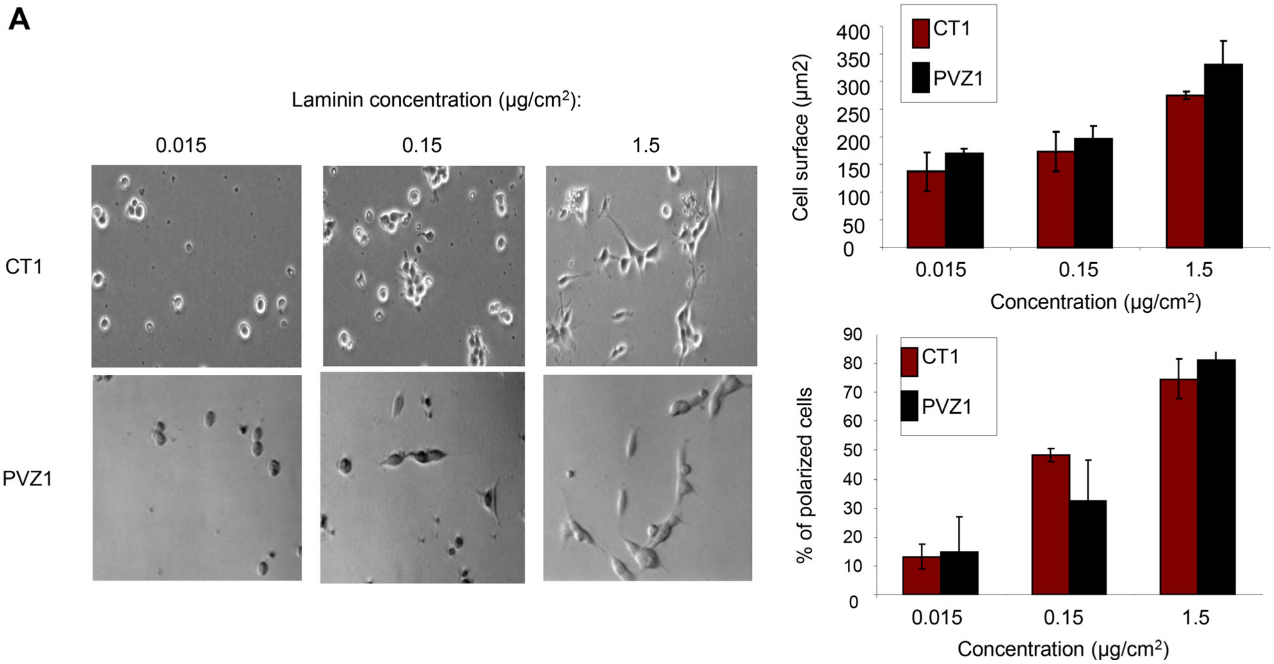

Supplementary Figure 2: CT and PVZ GSCs spread on laminin. (**A**) CT1 and PVZ1 GSCs were seeded on plates pre-coated with increasing laminin concentrations. Left: two hours after seeding, phase–contrast photographs were taken. Right: in each experiment, cell surface and percentage of polarized cells at least 30 individual cells were quantified as described in Material and Methods. Data is shown as means ± SD.

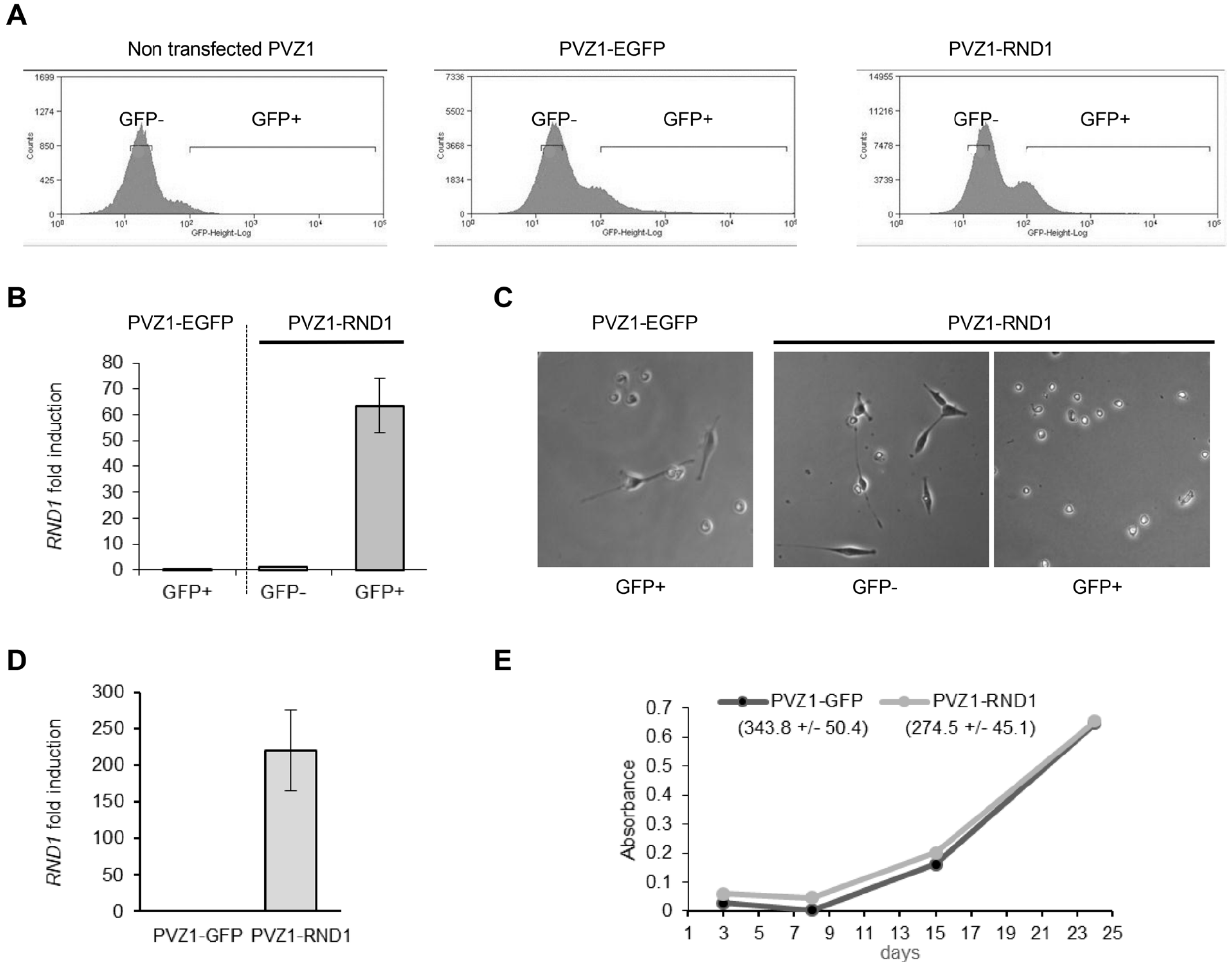

Supplementary Figure 3: (**A**) PVZ1 GSCs were transfected or not with pEGFP (PVZ1-EGFP) or pEGFP-RND1 plasmid (PVZ1-RND1). GFP- and GFP+ PVZ1 cells were then separated by cell sorting. (**B**) Analysis of *RND1* expression by RT-qPCR in GFP positive PVZ1-EGFP, GFP negative and positive PVZ1-RND1 cells. (**C**) GFP positive PVZ1-EGFP, GFP positive and negative PVZ1-RND1 cells were seeded on laminin, then allowed to spread for 3 h. Phase-contrast photographs were taken under ×10 magnification. (**D**) PVZ1 cells were stably transduced with lentiviral particles that contain a plasmid that allows the expression of *RND1* (PVZ1-RND1) or GFP (PVZ1-GFP). The expression of RND1 in PVZ1-RND1 and PVZ1-GFP cells was analyzed by RT-qPCR. Data is shown as fold induction means (±SEM) from 4 experiments. (**E**) Viability of PVZ1-GFP and PVZ1-RND1 cells was analyzed by a WST-1 assay. One representative experiment is shown. The figures in the brackets represent the means of proliferation rate (±SEM) from 3 experiments performed in triplicate.

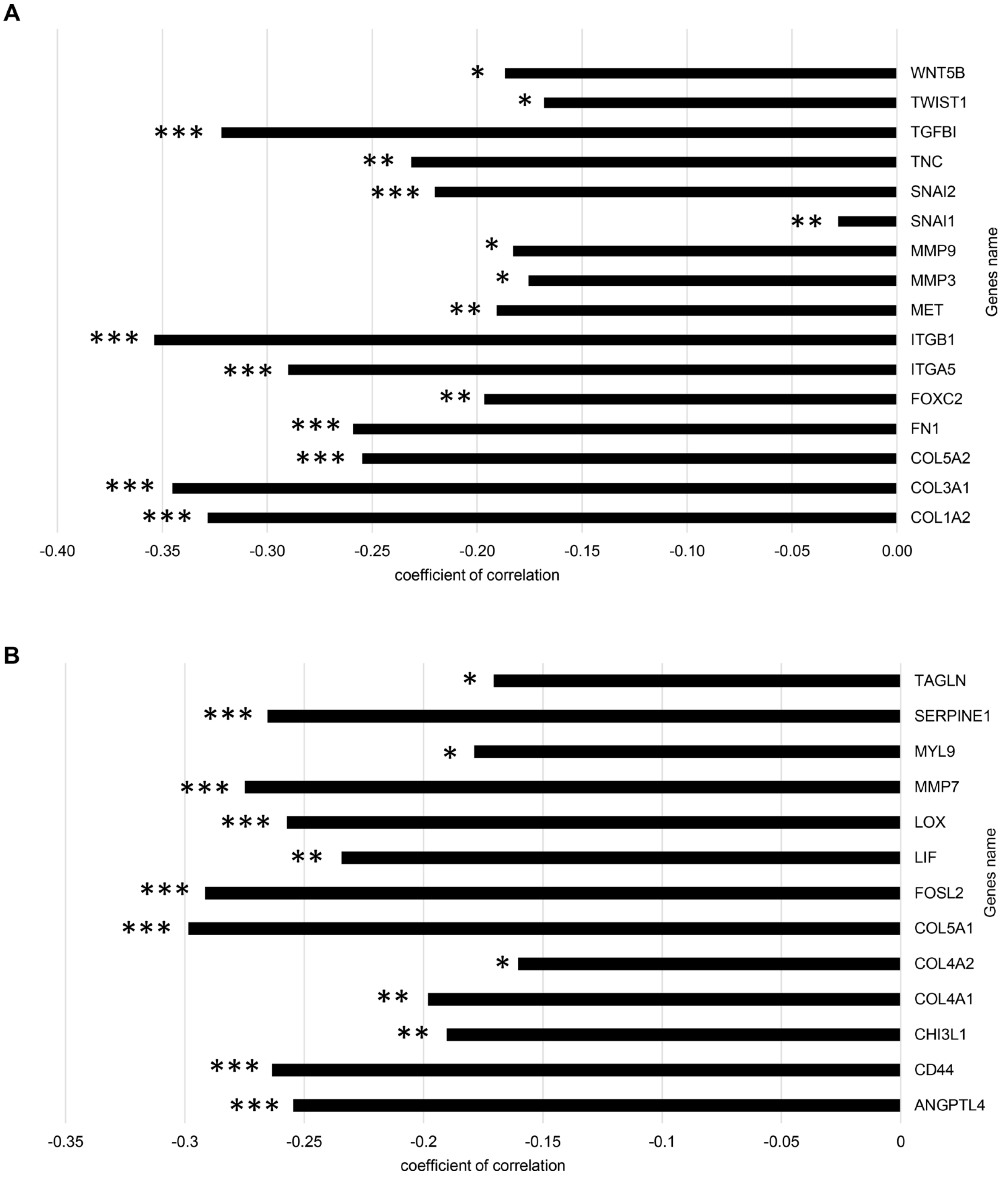

Supplementary Figure 4: *RND1* expression is inversely correlated with mesenchymal gene expression. The expression of *RND1* gene and mesenchymal genes was analyzed in glioblastoma patients using TCGA. The analysis concerns common mesenchymal genes (in **A**), and specific glioblastoma mesenchymal genes (in **B**). $^{*}p < 0.05$, $^{**}p < 0.01$, $^{***}p < 0,001$ by Spearman's coefficient test.

Supplementary Table 1: Raw data for limiting dilution assays (numbers in the table are percentage of positive wells)

Number of GSCs per well	1	2	4	8	16	31	62	125	250
CT1	12.5 ± 2.4	16.7 ± 2.4	31.9 ±10.8	59.7 ±13.9	72.2 ± 5.6	81.9 ±2.8	91.7 ± 4.2	100.0 ± 0.0	100.0 ± 0.0
PVZ1	16.6 ± 4.2	36.1 ±5.0	56.9 ± 8.4	76.4 ± 7.7	98.6 ± 1.4	100.0 ± 0.0	100.0 ± 0.0	100.0 ± 0.0	100.0 ± 0.0
CT2	27.1 ± 10.6	29.2 ±6.1	44.8 ±10.0	68.8 ± 11.6	86.5 ± 10.9	91.7 ± 5.9	97.9 ± 2.1	100.0 ± 0.0	100.0 ± 0.0
PVZ2	16.7 ± 7.4	20.8 ±7.4	40.6 ± 6.2	66.7 ± 9.5	89.6 ± 6.0	91.7 ± 3.8	100.0 ± 0.0	100.0 ± 0.0	100.0 ± 0.0

Supplementary Table 2: List of genes differentially expressed in CT and PVZ GSCs. See Supplementary_Table_2

Supplementary Table 3: List of non-protein coding RNAs differentially expressed in CT and PVZ GSCs

miRNA	small nucleolar	LINC	antisense	pseudogene	intronic transcript
miR3117	*sn RNA C/D box 116-25*	*LINC01344*	*AQP4*	*5S ribosomal pseudogene*	*ARHGEF9 intronic transcript 1*
miR4500 HG	*sn RNA C/D box 116-11*	*LINC01350*	*C3orf67*	*amine oxidase, copper containing 4, pseudogene*	*FTO-IT1*
miR491		*LINC00619*	*CNOT10*	*RNA, U6 small nuclear 1137, pseudogene*	
miR4265		*LINC00400*	*DNAJC9*	*RNA, U6 small nuclear 1210, pseudogene*	
miR4473		*LINC00642*	*MEOX2*	*RNA, U6 small nuclear 175, pseudogene*	
miR670			*MLIP*	*U2 small nuclear 22, pseudogene*	
			NLGN1	*U6atac pseudogene*	
			PABPC1L2B	*U6 small nuclear 661, pseudogene*	
			SAP30L	*U6 small nuclear 352 pseudogene*	
			USP12	*7SK small nuclear pseudogene 253*	
			ZNF790	*7SK small nuclear pseudogene 124*	
				U7 small nuclear 65 pseudogene	
				7SK small nuclear pseudogene 283	
				U6 small nuclear 1048 pseudogene	
				U6 small nuclear 1233 pseudogene	
				GOLGA8S	
				U2 small nuclear 6 pseudogene	
**miR3926−1**		**LINC00652**	**PTPRD**	**Ro-associated Y1 pseudogene**	
**miR4273**			**FGF14**	**7SK small nuclear pseudogene 259**	
			**FUT8**		
			**FOXN3**		

In grey and italics: down-regulated genes in PVZ GSCs; in black and bold letters: up-regulated genes in PVZ GSCs.

Supplementary Table 4: Patient baseline characteristics

	Patients with a lower *RND1* expression (*n* = 26)	Patients with a higher *RND1* expression (*n* = 158)	*P* value
Age (years)			
Age <60	13 (50.0%)	93 (58.9%)	$p = 0.3969$
Age >60	13 (50.0%)	65 (41.1%)	
G-CIMP status			
G-CIMP	1 (3.8%)	16 (10.5%)	$p = 0.4737$
Non G-CIMP	25 (96.2%)	137 (89.5%)	
Missing	0	5	
Karnofsky index			
<70	4 (15.4%)	27 (17.1%)	$p = 1.0000$
>70	22 (84.6%)	131 (82.9%)	
Initial pathologic diagnosis			
Excisional biopsy	1 (3.8%)	29 (18.7%)	$p = 0.0839$
Tumor resection	25 (96.2%)	126 (81.3%)	
Missing	0	3	
Molecular subtype			
Classical	6 (23.1%)	46 (29.1%)	$p = 0.0002$
Mesenchymal	17 (65.4%)	37 (23.4%)	
Neural	1 (3.8%)	30 (19.0%)	
Proneural	2 (7.7%)	45 (28.5%)	

Supplementary Table 5: List of signaling pathways from KEGG

Pathway	*p* value ZE	Number of common genes	Common genes
ECM RECEPTOR INTERACTION	1.32E-09	*18/84*	COL6A2 COL6A3 COL5A2 COL6A1 ITGA4 ITGA5 COL5A1 COL3A1 ITGB1 LAMC1 COL1A1 LAMA5 LAMB1 COL1A2 CD44 THBS3 SDC1 THBS1
FOCAL ADHESION	1.05E-07	*25/201*	LAMC1 LAMA5 LAMB1 FLNA MET THBS3 THBS1 ACTN1 CAV2 CAV1 COL6A2 COL6A3 COL5A2 COL6A1 ITGA4 ITGA5 COL5A1 COL3A1 ITGB1 COL1A1 COL1A2 VCL SHC1 RAC2 PPP1CA
LYSOSOME	1.72e-04	*14/121*	IGF2R CTSC CD164 CTSB TCIRG1 GNS DNASE2 M6PR GUSB GLA NPC2 GLB1 HEXB CTSA
PROTEIN EXPORT	1.77e-03	*5/24*	SRPR HSPA5 OXA1L SEC11A SEC61A1
O GLYCAN BIOSYNTHESIS	4.93E-03	*5/30*	GALNT4 GALNT7 GCNT1 GALNT2 ST3GAL1
APOPTOSIS	1.69E-03	*10/88*	CASP8 CASP7 MYD88 TNFRSF1A NFKB1 IL1RAP IL1R1 IRAK1 TRADD FAS
N GLYCAN BIOSYNTHESIS	2.91e-02	*5/46*	RPN2 MGAT2 GANAB DDOST MAN2A1
COMPLEMENT AND COAGULATION CASCADES	1.51e-02	*7/69*	PLAUR PLAU TFPI CD59 CD46 PROS1 SERPINE1
GLYCOSPHINGOLIPID BIOSYNTHESIS GANGLIO SERIES	1.75e-02	*3/15*	GLB1 ST3GAL1 HEXB
GLYCOSAMINOGLYCAN DEGRADATION	7.26e-03	*4/21*	GLB1 GUSB GNS HEXB
GLYCOSPHINGOLIPID BIOSYNTHESIS GLOBO SERIES	1.44e-02	*3/14*	GLA ST3GAL1 HEXB
REGULATION OF ACTIN CYTOSKELETON	7.56e-03	*16/216*	ARPC5 ARPC1B PFN1 MYH9 ACTN1 IQGAP1 ITGA4 ITGA5 SLC9A1 ITGB1 VCL RAC2 PPP1CA ARPC2 RRAS GNG12
ADHERENS JUNCTION	7.14e-03	*8/75*	SNAI2 ACTN1 RAC2 IQGAP1 VCL TGFBR2 MET PTPN1

Supplementary Table 6: Details about genes that form the 6 gene prognostic signature

Gene symbol	Gene name	Protein description	Lasso coefficient	BSS
ITGA5	Integrin alpha 5	In association with integrin beta 1, they form a cell adhesion protein	0.18785091	0.793
COL5A1	Collagen Type V Alpha 1 Chain	Extracellular matrix protein	0.07963124	0.552
COL3A1	Collagen Type III Alpha 1 Chain	Extracellular matrix protein	−0.09632951	0.617
LAMC1	Laminin subunit gamma 1	Extracellular matrix protein	0.17416926	0.528
COL1A2	Collagen Type I Alpha 2 Chain	Extracellular matrix protein	−0.04758932	0.527
MET	MET	HGF Receptor	0.07033914	0.602

Supplementary Table 7: Extracellular matrix proteins

Protein	Concentration	Reference	Suppliers
Laminin 111	1.5 µg/cm^2	L2020	Sigma
Fibronectin	1.5 µg/cm^2	354008	BD Biosciences
Vitronectin	1.5 µg/cm^2		Prepared as described in Yatohgo *et al*., Cell Struct Funct, 1988.

Supplementary Table 8: Primary antibodies

Marker	Antibodies	Reference	Suppliers	Application
**A2B5**	IgM mouse monoclonal antibody– APC	130-093-582	Miltenyi	Flow cytometry
**α3 integrin**	IgG1 mouse monoclonal antibody	MAB1952	Millipore	Function-blocking antibody
**α6 integrin**	IgG2a,K rat monoclonal antibody	MAB1378	Millipore	Function-blocking antibody
**α6 integrin**	IgG2a,K-PE rat monoclonal antibody-PE	12-0495	eBiosciences	Flow cytometry
**β1 integrin**	IgG1 mouse monoclonal antibody	MAB2253	Millipore	Function-blocking antibody/ Flow cytometry
**β4 integrin**	IgG1 mouse monoclonal antibody	Clone AA3	Gift from Dr Quaranta	Function-blocking antibody
**β4 integrin**	IgG2b mouse monoclonal antibody-AF488	FAB4060G	R&D Systems	Flow cytometry
**CD133**	IgG2B mouse monoclonal antibody-PE	130-090-853	Miltenyi	Flow cytometry
**GFAP**	mouse monoclonal	MAB 5628	Millipore	Immunofluorescence
**NESTIN**	mouse monoclonal	MAB5326	Millipore	Immunofluorescence / Immunohistochemistry
**NESTIN**	IgG1 mouse monoclonal antibody - Fluorescein	IC1259F	R&D Systems	Flow cytometry
**SOX2**	Igg2A mouse monoclonal antibody– APC	IC2018A	R&D Systems	Flow cytometry
**SOX2**	mouse monoclonal	MAB4343	Millipore	Immunofluorescence
**β3 tubulin**	mouse monoclonal	MAB1637	Millipore	Immunofluorescence

Supplementary Table 9: Quantitative PCR primer sequences (Eurogentec)

Gene	Forward primer	Reverse primer
α3 integrin	5′ TGTGGCTTGGAGTGACTGTG 3′	5′ TCATTGCCTCGCACGTAGC 3′
α6 integrin	5′TGATCGAAATTCCTACCCTGATG 3′	5′TAATCACAGGCCGGGATCTG 3′
β2 microglobulin	5′ ACCCCCACTGAAAAAGATGA 3′	5′ ATCTTCAAACCTCCATGATG 3′
β1 integrin	5′ CAAAGGAACAGCAGAGAAGC 3′	5′ ATTGAGTAAGACAGGTCCATAAGG 3′
β4 integrin	5′ CTGTACCCGTATTGCGACT 3'	5′ AGGCCATAGCAGACCTCGTA 3′
CD133	5′ GCATTGGCATCTTCTATGGTT 3′	5′ CGCCTTGTCCTTGGTAGTGT 3′
GFAP	5′ GGCAAAAGCACCAAAGACGG3′	5′ GGCGGCGTTCCATTTACAAT3′
MAL	5′ CGCTGCCCTCTTTTACCTCAG 3′	5′ GAAGCCGTCTTGCATCGTGAT 3′
Olig1	5′-AGGTAACCAGGCGTCTCACAGT-3′	5′ CGGTACTCCTGCGTGTTAATGA 3′
Olig2	5′-CAGAAGCGCTGATGGTCATA-3′	5′ TCGGCAGTTTTGGGTTATTC 3′
OMG	5′ TAGGGACTCCATGTTCTACCCA 3′	5′ TCTGCATCCCACTTACAGTGA 3′
Nestin	5′ ATCGCTCAGGTCCTGGAAGG 3′	5′ AAGCTGAGGGAAGTCTTGGAG 3′
Rnd1	5′ GCAAGTGTTAGCGAAGGATTG 3′	5′ GCAGAGTGGACGGACATTATC 3′
Sox2	5′ GCACATGAACGGCTGGAGCAACG 3′	5′TGCTGCGAGTAGGACATGCTGTAGG 3′
β3 tubulin	5′GCTCAGGGGCCTTTGGACATCTCTT3′	5′ TTTTCACACTCCTTCCGCACCACATC3′

Supplementary Table 10: Percentages of GSCs in neurospheres

	% of GSCs in neurospheres
CT1	60.9 ± 6.5
PVZ1	75.1 ± 8.9
CT2	56.5 ± 6.8
PVZ2	64.5 ± 1.7

Supplementary Table 11: References of the selected datasets

Study number	Serie number	References	Fold	*p* value
A	GSE4381	Yamanaka R *et al*. Oncogene. 2006. PMID: 16652150	–2,43	1.10E-09
B	GSE19728	Zhongyu L *et al*. Molecular neurobiology. 2012. PMID: 23135747	–2,43	0.0493
C	GSE61335	Joy A *et al*. PLoS One. 2014. PMID: 24984002	–2,14	0.000000000049
D	GSE15824	Grzmil M *et al*. Cancer Res. 2011. PMID: 21406405	–1,84	0.0046
E	GSE4290	Sun L *et al*. Cancer Cell 2006. PMID: 16616334	–1,86	3.90E-05
F	GSE66354	Donson M A. UC Denver	–1,84	0.0029
G	GSE22866	Etcheverry A *et al*. BMC Genomics. 2010. PMID: 21156036	–5,96	2.60E-11
H	GSE10878	de Tayrac M *et al*. Genes Chromosomes Cancer. 2009. PMID: 18828157	–1,72	3.70E-05
I	GSE15209	Pollard SM *et al*. Cell Stem Cell 2009.	–1,37	0.0044
J	GSE12657	Margareto J *et al*. J Mol Neurosci. 2007.	–1,76	0.0003
K	TCGA	Cancer Genome Atlas Research Network. Nature. 2008. PMID: 18772890; 20129251; 20399149; 20838435	–1,46	0.0016
L	GSE14805	Hodgson JG *et al*. Neuro Oncol 2009. PMID: 19139420	–3,83	3.30E-05
M	GSE68848	REMBRANDT	–1,75	0.0005
